# Clinical drug trials in general practice: how well are external validity issues reported?

**DOI:** 10.1186/s12875-017-0680-7

**Published:** 2017-12-29

**Authors:** Anja Maria Brænd, Jørund Straand, Atle Klovning

**Affiliations:** Department of General Practice, Institute of Health and Society, Faculty of Medicine, University of Oslo, Postbox 1130 Blindern, N-0318 Oslo, Norway

**Keywords:** General practice, Clinical trials, External validity, Applicability, Generalizability, Eligibility determination

## Abstract

**Background:**

When reading a report of a clinical trial, it should be possible to judge whether the results are relevant for your patients. Issues affecting the external validity or generalizability of a trial should therefore be reported. Our aim was to determine whether articles with published results from a complete cohort of drug trials conducted entirely or partly in general practice reported sufficient information about the trials to consider the external validity.

**Methods:**

A cohort of 196 drug trials in Norwegian general practice was previously identified from the Norwegian Medicines Agency archive with year of application for approval 1998–2007. After comprehensive literature searches, 134 journal articles reporting results published from 2000 to 2015 were identified. In these articles, we considered the reporting of the following issues relevant for external validity: reporting of the clinical setting; selection of patients before inclusion in a trial; reporting of patients’ co-morbidity, co-medication or ethnicity; choice of primary outcome; and reporting of adverse events.

**Results:**

Of these 134 articles, only 30 (22%) reported the clinical setting of the trial. The number of patients screened before enrolment was reported in 61 articles (46%). The primary outcome of the trial was a surrogate outcome for 60 trials (45%), a clinical outcome for 39 (29%) and a patient-reported outcome for 25 (19%). Clinical details of adverse events were reported in 124 (93%) articles. Co-morbidity of included participants was reported in 54 trials (40%), co-medication in 27 (20%) and race/ethnicity in 78 (58%).

**Conclusions:**

The clinical setting of the trials, the selection of patients before enrolment, and co-morbidity or co-medication of participants was most commonly not reported, limiting the possibility to consider the generalizability of a trial. It may therefore be difficult for readers to judge whether drug trial results are applicable to clinical decision-making in general practice or when developing clinical guidelines.

## Background

When reading a report of a clinical trial, it should be possible to judge whether the results are relevant for the patients in your own practice, namely: *“Can I apply the results of this trial to my patients?”* [1, 2]. The terms external validity, applicability, representativeness, and generalizability are used quite synonymously to denote to which populations or settings the effect of a trial may be generalised or extrapolated [1, 3]. However, this difficult judgement is far too often left for each clinician to decide. By contrast, *“Can I trust the results?”*, is a question of internal validity, where helpful tools have been developed and are commonly used, e.g. The Cochrane Group’s Risk of Bias tool [4].

Standards for reporting clinical trials have been developed during the past 20 years, resulting in the CONSORT checklist [5], which is now widely used [6]. However, although there are items in the CONSORT checklist connected to external validity, the main focus is on adequate reporting of trial elements affecting the internal validity of a trial, i.e. the extent to which the design and conduct of a trial eliminates the possibility of bias [5]. The CONSORT extension for pragmatic trials from 2008 elaborates the CONSORT checklist with recommendations for the reporting of pragmatic trials, i.e., trials designed for maximising applicability to usual care settings, thereby complementing the CONSORT statement on external validity issues [7]. The intention of this addition to the CONSORT checklist is to guide authors in reporting factors affecting external validity of trials.

Patients eligible for inclusion in randomised controlled trials (RCTs) are too often not broadly representative of patients encountered in everyday practice [8]. This has been demonstrated for several therapeutic areas, including diabetes [9], chronic obstructive pulmonary disease (COPD) [10], asthma [11], infectious diseases [12], and depression [13]. Patient samples in efficacy or explanatory trials (trials under optimal conditions) are generally more homogenous with less co-morbidity and a lower risk of complications compared with patients included in effectiveness or pragmatic trials (trials under real-world conditions) [14]. Higher-risk patients often account for most of the treatment benefit in trials; therefore, subgroups of patients might have important differences in terms of treatment benefits [15].

Judgements regarding the external validity of a trial depend on the reporting of key characteristics of adequate information regarding participants in the trial, trial settings, the treatments tested and the outcomes assessed [5]. This judgement has been described as a “complex reflection in which prior knowledge, statistical considerations, biological plausibility and eligibility criteria all have place” [16]. Several checklists for considering external validity have been proposed and systematically reviewed [17]. None of the identified checklists were based on empirical data, and those based on literature reviews were not considered to provide a clear connection between the references and checklist items [17]. The authors concluded that there exists no current consensus regarding how to assess external validity [17]. Because external validity depends on the context, there might be inescapable problems with designing a universal checklist [18]. To judge the external validity of a trial, adequate reporting of the setting, intervention and participants is paramount; however, reporting is often insufficient [19, 20].

Our aim was to assess whether articles with published results from a cohort of general practice drug trials gave sufficient information about each trial to consider important aspects of the external validity relevant for general practice. Specifically, the objectives were to assess the reporting of trial settings, the selection of patients, key characteristics of randomised patients, choice of outcome measures, and adverse effects of treatment. We aimed to explore the change in reporting during the time period for these variables. We also present a case study to illustrate clinical characteristics of patients included in the type 2 diabetes trials in the cohort.

## Methods

### Cohort of general practice drug trials

In Norway, all clinical pharmaceutical trials require approval from the Norwegian Medicines Agency (NoMA), a national regulatory authority for new and established drugs. We hand searched the NoMA paper-based (i.e. not electronic) archive, and identified protocols for trials planned to be conducted in general practice for the 10-year period 1998–2007, before the introduction of a new archive system. Trials were included in the cohort if any of the clinical investigators was a general practitioner (GP). The identification and main characteristics of the trials have been described previously [21]. The trial cohort included 196 trials, of which 189 were industry-initiated and 182 were multinational, with a total planned sample size of over 330,000 patients [21]. The median recruitment target was 673 patients internationally (range 8–31,000). A majority of the 151 trials took place in a combination of general practice and specialist care settings. According to the protocols, the trials were planned to be completed between 1998 and 2012. Diabetes drugs were the most frequent drug group, representing 20% of the trials. We subsequently searched for publications from this cohort of trials in MEDLINE, Embase, and the Cochrane Central Register of Controlled Trials (CENTRAL), and identified that 135 trials had results published in a journal article [22]. The most recent search for publications was performed December 2015; otherwise, there was no exclusion related to publication date. Many trials had several publications, a total of 285 journal articles were connected to the trials, and 134 of these were defined as main journal articles presenting results [22]. In the present paper, we describe how these 134 articles with publication year span of 2000–2015 reported issues relevant for judging external validity. If an issue was not reported in the main article, we checked whether it was reported in an online appendix or in any of the other journal articles we had identified from the same trial.

### Data extraction

We developed a data extraction form in a web-based database with written instructions for coding, and then pilot tested it with all three of the present authors.

One author extracted data for all articles regarding the methodological characteristics of each trial. The articles were screened manually for information in the relevant sections; in addition, searches for the relevant search terms were performed using the PDF search option in EndNote X7 bibliographic software. Doubt regarding the coding was resolved by consensus. As it was not feasible for two authors to extract data from all articles, a random sample of 66/134 (49%) trials was selected, data were extracted independently by another author, and kappa statistics for agreement between the two assessors was calculated. We used an Internet based random number generator (www.random.org) to select the random sample based on the trial identification number. Any discrepancies were resolved by discussion and consensus.

We shortened and modified Rothwell’s extensive panel of issues potentially affecting external validity for the coding of publication characteristics and methodology (Table 1) [1]. We chose to include the aspects we considered most relevant for general practice, and also feasible for assessment across the wide range of therapeutic areas.

**Table 1:** Reporting of major issues that potentially affect external validity according to Rothwell [1]

• Setting of the trial	
• Selection of patients	
• Characteristics of randomised patients	
• Differences between the trial protocol and routine practice	
• Outcome measures and follow-up	
• Adverse effects of treatment	

Extracted data included whether the authors reported the clinical setting of the trial, namely whether trial sites were in general practice/family practice/family medicine/primary care or in hospitals/specialist care. We recorded whether the number of patients screened, i.e., assessed for eligibility before inclusion in the actual trial, was reported. Furthermore, we recorded the numbers of patients who declined, enrolled, and completed the trial.

We determined the primary outcome of the trials as specified in the articles, or, if not specified, we defined the primary outcome as the outcome used in the power calculations, if reported. The primary outcome was classified as clinical, patient-reported, surrogate, costs or other. Clinical outcomes were defined as morbidity or mortality, and measurements of patient survival or function such as incidence of disease or hospitalisation [23]. Patient-reported outcomes included clinical scales (grading of symptoms) and other quantifications of subjective symptoms or complaints. Surrogate outcomes were defined as intermediate outcomes intended to substitute for a clinical endpoint and predict benefit or harm, e.g. HbA1c, cholesterol levels or blood pressure [23]. If several endpoints were mentioned among primary outcomes, we recorded the most clinically relevant outcome. We also recorded whether any of the other presented outcomes were patient-relevant, measured quality of life or costs or if no trial outcomes were in any of the mentioned outcome categories.

For all trials, we recorded whether eligibility criteria (defined as clinical inclusion or exclusion criteria) were reported. We also recorded whether the articles reported the co-morbidity, co-medication, and race/ethnicity of participants.

As a case study, we investigated diabetes trials in more detail because they made up the largest group of tested drugs in the cohort. Specifically, we looked at patient characteristics in RCTs of type 2 diabetes. We recorded details regarding eligibility criteria and the key baseline characteristics of trial participants to discuss in the light of other published data on type 2 diabetes patients in general practice.

For all trials, we recorded whether clinical details of adverse events in the trial were reported.

### Statistical analyses

Data were analysed using descriptive statistics. We used chi-square tests for trends in reporting over time during the publication years 2000–2015 [24], with *p* < 0.05 considered as statistically significant. We calculated the kappa measure of agreement between raters, and kappa 0.61–0.8 was considered to represent good agreement [24]. Statistical analyses were performed using IBM SPSS Statistics for Windows (version 24), and chi-square tests for trends were conducted with GraphPad Prism 7.

## Results

Of the 134 trials, 125 (93%) were randomised and 101 (75%) were blinded. For 85 trials, information regarding the trial phase [14] was available; six (5%) were phase 2, 55 (41%) were phase 3, and 24 (18%) were phase 4.

### Reporting of trial setting

**Fig. 1:**
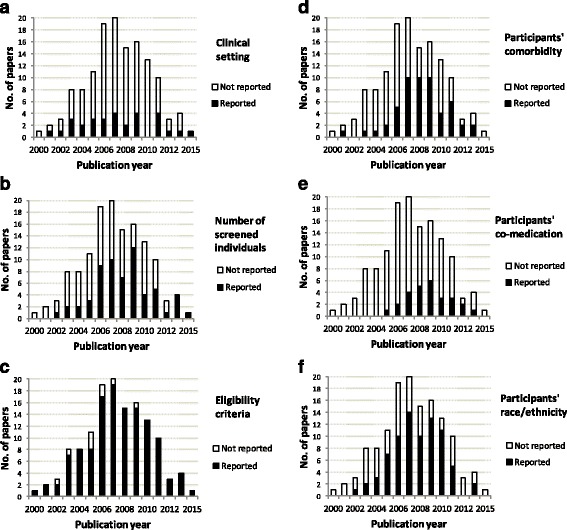
Reporting of external validity issues in 134 drug trials in general practice published 2000–2015. **a**–**f** shows the development over time of reporting of (**a**) clinical setting, (**b**) screened individuals, (**c**) eligibility criteria, (**d**) participants’ co-morbidity, (**e**) co-medication, and (**f**) race/ethnicity

The clinical setting of the trial was described in 30 (22%) of the trials (Table 2). The reporting of setting did not change during the time period (Fig. 1a). The clinical setting was described in a higher proportion of trials with a general practice setting only, 14/29 (48%), compared with trials with a mixed setting, 16/105 (15%) (*p* < 0.001, chi-square test). This was the only variable with a significant difference between reporting for a general practice only vs. a mixed setting.

**Table 2:** External validity items reported in 134 drug trials in general practice

	Trials reporting data		Change in reporting over time
	n	%	*p*-value*
Total	134	100	
Setting of the trial			
Country	106	79.1	0.12
Clinical setting	30	22.4	0.82
Trial sites	122	91.0	0.05
Number reported	109	81.3	
Names/affiliations listed	55	41.0	
Selection of patients			
Number of patients screened	61	45.5	**0.005**
Number of individuals who declined	24	17.9	0.12
Number of patients enrolled	134	100.0	
Number of patients completed	125	93.3	0.34
Eligibility criteria reported	125	93.3	**0.03**
Characteristics of randomised patients			
Comorbidity of included patients	54	40.3	**0.002**
Co-medication of included patients	27	20.1	**0.002**
Race/ethnicity of included patients	78	58.2	0.05
Outcome measures			
Primary outcome			
clinical	39	29.1	
patient reported	25	18.7	
surrogate	60	44.8	
cost	1	0.7	
other	7	5.2	
not clearly defined	2	1.5	
Surrogate outcomes only^a^	47	35.1	0.72
Adverse effects of treatment			
Details of adverse events	124	92.5	0.20

*Chi-square test for trend in reporting over publication years 2000–2015

^a^Includes primary and all other reported outcomes

### Reporting of patient selection

The number of patients screened was reported for 61 (46%) trials (Table 2). The proportion of articles reporting the number of screened patients increased during the time period (Fig. 1b). For nine trials, the number of patients completing the trial was not clearly reported; three of these were terminated prematurely, while the others did not clearly report this number. Power or sample size calculations were reported in 97 (72%) trials, whereas no power calculations were reported in 31 (23%); for six (4.5%) trials, we considered this as unclear or irrelevant.

Table 3 shows the reported selection of patients. The mean fraction of enrolled patients completing the trials was 0.83. The mean number needed to be screened to include one participant was 1.94; however, numbers available to calculate this were only available for 61 (46%) trials.

**Table 3:** Reporting of trial sites, selection of patients, recruitment and completing fractions

	Trials reporting data n (%)	Median^a^	IQR	Min-max	Mean	95% CI
Number of countries	106 (79)	10	7–15	1–45		
Number of trial sites	122 (91)	96	61–171	1–1315		
Number of patients screened	61 (46)	1284	615–2352	14–89,890		
Number of patients enrolled	134 (100)	910	503–1743	13–29,019		
Number of patients completed	125 (93)	648	379–1134	13–25,577		
Recruitment fraction	61 (46)	0.66	0.46–0.83	0.17–1.00	0.64	(0.58–0.70)
Number needed to be screened	61 (46)	1.52	1.20–2.20	1.00–5.84	1.94	(1.64–2.22)
Completing fraction 1	125 (93)	0.83	0.70–0.89	0.19–1.00	0.78	(0.75–0.80)
Completing fraction 2	59 (44)	0.53	0.38–0.70	0.13–0.93	0.53	(0.47–0.58)

Definition of terms:

*Recruitment fraction:* Proportion of screened people who enrol in the trial (all enrolled/all screened), *Number needed to be screened:* Number of people screened in order to enrol one participant (1/recruitment fraction), *Completing fraction 1:* Proportion of participants who completed the trial (all completed/all enrolled), *Completing fraction 2:* Proportion of screened people who completed the trial (all completed/all screened), *IQR:* Inter-quartile range. *CI:* Confidence interval

^a^Mean not calculated for frequencies because of skewed data

A run-in period with active treatment was a part of the trial design in 23 (17%) trials, whereas 95 (71%) did not have a run-in period with active treatment; for 16 (12%) trials, we characterised this to be unclear or irrelevant, e.g. because the trial was an extension of another trial.

Specific eligibility criteria were reported in 93% of the trials (Table 2). The proportion reporting eligibility criteria improved over the time period (Fig. 1c).

### Reporting of characteristics of randomised patients

Co-morbidity of trial participants was reported in 54 (40%) trials and co-medication in 27 (20%); the reporting increased over the time period (Table 2, Fig. 1d–f).

In the case study of type 2 diabetes trials, the age groups were restricted to adults over 18 years for 83% of the trials, and 65% restricted participation to patients <80 years (Table 4). No trial excluded female patients, but six (26%) had exclusion criteria related to pregnancy, contraception, and/or lactation. Exclusion criteria related to co-morbidity were reported for 21 (91%) trials; the most common was exclusion of patients with renal dysfunction. Exclusion criteria related to concurrent medication use was reported in 35% of the trials. No trial reported race/ethnicity of patients as an exclusion criterion. Key baseline data of the participants of the trials and reporting of co-morbidity, co-medication or race/ethnicity are shown in Table 4 and compared with other published data regarding patients with type 2 diabetes from a nationwide Scottish cohort [9], a population based Norwegian study (HUNT-2) [25] and a Belgian general practice study [26].

**Table 4:** Reported eligibility criteria and key baseline characteristics of type 2 diabetes randomised controlled trials (*n* = 23)

	Exclusion criteria in total of 23 trials	Baseline data of included patients. Mean (CI)	Data from other population/GP studies for comparison		
Exclusion criteria			Scotland^a^	Belgium^b^	Norway^c^
Age		58.2 years (56.3–60.1)	66.3	58	62.4
< 18	19				
< 21	1				
< 40	1				
< 65	1				
No lower limit reported	1				
> 70–79	10				
> 80	5				
No upper limit reported	8				
Gender		54.2% male (52.4–56.0)	54.3	51.9	49.9
Females	0				
Pregnancy	3				
Lack of contraception use	3				
Breastfeeding	1				
HbA1c		8.1% (7.85–8.35)	7.4	7.1	7.7
< 6	1				
< 6.5	4				
< 7	8				
< 7.5	5				
No lower limit reported	5				
> 9	2				
> 10–10.9	11				
> 11	4				
> 12	2				
No upper limit reported	4				
BMI		30.6 kg/m^2^ (30.1–31.1)	31.4	31	29.7
< 18	1				
< 22–23	4				
> 35	3				
> 40–45	6				
No BMI exclusion criteria reported	14				
Medical comorbidities		7 trials reported participants’ comorbidity			
Coronary heart disease	14				
Heart failure	9				
Stroke	4				
Renal dysfunction	17				
Liver dysfunction	14				
Other type of diabetes	13				
Previous or suspected drug intolerance	3				
Other disease	7				
Medication related		5 trials reported participants’ co-medication			
Concurrent medication	8				
Race/ethnicity	0	19 trials reported participants’ race/ethnicity			

Randomised controlled trials for type 2 diabetes drugs: DPP4-inhibitors (*n* = 12), insulins (*n* = 5), PPAR-agonists (*n* = 2), thiazolidinedones (*n* = 2) and others (*n* = 2). The numbers of trials reporting different key eligibility criteria are presented with mean baseline characteristics of included patients. Baseline characteristics are compared to published data from three population based or general practice studies

Data from other studies:

^a^Scotland: Nationwide diabetes clinical database [9]

^b^Belgium: General practice morbidity registration [26]

^c^Norway: Population based study HUNT-2 [25]

### Reporting of outcome measures and follow-up

The primary outcome of the trial was a surrogate outcome in 45% of the trials and a clinical outcome in 29%; 35% of the trials reported a surrogate outcome only, including all secondary outcomes (Table 2). The analyses were done according to the intention-to-treat (ITT) principle in 26 trials (19%), modified ITT analyses were reported in 82 (61%), and no ITT analyses were reported in 17 trials (13%); this was unclear or irrelevant for nine trials (6.7%).

### Reporting of adverse effects of treatment

Clinical details of adverse effects of treatment were reported in 93% of the trials (Table 2). Only two articles did not report any numbers of adverse events, while eight reported the numbers without specifying which adverse events the patients had experienced.

The inter-rater reliability for assessing the methodological characteristics of 49% of the randomly selected articles was good, with a mean kappa of 0.70 for all variables.

## Discussion

We investigated the reporting of issues relevant for judging the external validity of a 10-year cohort of drug trials conducted in general practice. Important issues potentially affecting external validity were frequently not reported. A minority of the articles reported the clinical setting, the number of patients screened before enrolment and co-medications and co-morbidities.

### Setting of the trials

We found that reporting the clinical setting of a trial was frequently omitted because it was only reported in about 20% of the articles, and we did not observe any improvement in this reporting over the time period. The scarce reporting of the clinical setting is inconsistent with the recommendation in the CONSORT criteria [5]. Information about the setting is considered crucial for assessment of the applicability of a trial [1, 7, 16, 27, 28]. Description of the setting and participant eligibility criteria were a collective checklist item in the 2001 version of the CONSORT statement [5]. In the 2010 version, this was split into two sub-items for better interpretation [6]. Both the 2010 version of the CONSORT statement and the CONSORT extension for pragmatic trials were issued during the time period when the articles were published [7]. A study of NICE guidelines aimed at primary care showed that a substantial proportion of relevant recommendations were derived from studies that were not conducted in primary care. The investigators also found surprising difficulty in determining the setting because it was often only vaguely reported, despite the CONSORT guidelines recommendations [29]. Similarly, systematic reviews often do not provide data regarding the clinical setting of trials included, and often do not discuss whether results are applicable for primary care [30]. Moreover, the authors of systematic reviews aimed for primary care should report external validity issues relevant for primary care [30, 31].

### Selection of patients: Patient flow

We found that less than half of the articles reported patient selection before randomisation, but this omission decreased during the time period. Compared with similar studies, we found a considerably lower proportion of articles reporting the number of patients screened for eligibility, but a comparable good reporting of participant flow after inclusion. Jones et al. assessed RCTs published in primary care journals 2001–2004 and found that 70% reported the number of individuals assessed by investigators for eligibility, while all reported the actual number recruited [32]. Few of these trials were industry funded, and few were drug trials [32]. In high-impact general medical journals, 52–60% of RCTs published during 1999–2000 and 2004 reported the numbers of patients screened for eligibility [33, 34]. The CONSORT statement recommends reporting of the number of persons assessed for eligibility if available, but this is regarded as less important than the participant flow after inclusion [5, 27]. However, the CONSORT extension for pragmatic trials explicitly recommends reporting of the screening process [7].

### Selection of patients: Eligibility criteria

More than 90% of the articles in our cohort of trials reported specific eligibility criteria. The proportion reporting eligibility criteria improved during the time period. Our results are consistent with the findings of van Spall et al.; of RCTs published in major medical journals, they found that 12% of the trials did not report exclusion criteria [35]. Reporting of eligibility criteria has been emphasised as particularly important [1, 7, 16, 28], and it is also one of the CONSORT items, however previously often not reported adequately [5]. Blümle et al. compared the prespecified eligibility criteria in trial protocols submitted to a German ethics committee with the eligibility criteria later presented in journal articles, and they found that trial eligibility criteria were often incompletely or inadequately reported in journal articles [36]. The discrepancies they found might hamper a proper assessment of the applicability of published trial results [36]. In our cohort of trials, we investigated whether eligibility criteria were reported in published articles from the trials only, but did not examine the eligibility criteria reported in the trial protocols.

### Characteristics of randomised patients: Multi-morbidity and co-medication

We found that only 40% of articles reported co-morbidity of the participants. This is concerning, as multi-morbidity is common among patients in general practice [37], and is strongly related to adverse drug events [38]. Whether patients with multi-morbidity are included or excluded in a trial should be reported, but is often omitted, even in hypertension trials relevant for general practice populations with a high prevalence of co-morbid conditions [20]. An analysis of published RCTs showed that common medical conditions and commonly prescribed medications were frequent reasons for exclusion, but often poorly justified [35]. Drug trials were more likely than other trials to exclude individuals because of concomitant medication use, co-morbidities or female gender [35]. In a review of methodological papers on the representativeness of RCT samples, patients enrolled in RCTs in cardiology, mental health and oncology generally had fewer co-morbidities than real-world patients [8]. A study of RCTs registered at ClinicalTrials.gov 2014–2015 showed that more than three-quarters of trials for patients with chronic conditions excluded patients with multi-morbidity, suggesting that this remains a highly relevant issue [39].

Only about 20% of articles from our cohort of trials reported concomitant drug use. We find this concerning because polypharmacy is common in general practice. However, the reporting of co-medication improved over time. In clinical practice, dealing with polypharmacy represents a major challenge. Potential interactions between drugs are not typically considered in clinical guidelines, even though potentially serious drug interactions are common when applying several clinical guidelines for frequently co-morbid conditions [40]. GPs find it challenging to treat patients with multi-morbidity using complex medication regimens and disease-specific guidelines that do not consider multi-morbidity [41].

### Case: Eligibility criteria and key baseline characteristics in type 2 diabetes trials

Of the diabetes trials in our cohort, none excluded female patients, but co-morbidity or concomitant medication use were frequent reasons for exclusion. Comparing the baseline characteristics of participants in trials included in the present study with cohort studies from Scotland [9], Belgium [26] and Norway [25], participants in the trials were younger than Scottish and Norwegian patients with type 2 diabetes and had a higher baseline HbA1c than patients in all three previous cohort studies. Otherwise, the baseline characteristics were comparable to the population-based data. Saunders et al. found that the external validity of other large diabetes trials was limited compared with the population-based Scottish patient cohort, in particular, trial participants were generally younger than the general patient population [9]. In a U.S. national survey, potential treatment effect modifiers, i.e. specific clinical diseases or conditions with a well-described mechanism for treatment effect modification, were found to be highly prevalent, especially among older adults with type 2 diabetes, with the potential to alter treatment effects in everyday practice compared with clinical trial populations [42]. Only a few articles in our present cohort reported the co-morbidity and co-medication of participants, limiting the assessment of this important aspect.

### Outcome measures and follow-up

We found that nearly 50% of the trials had a surrogate outcome as their primary outcome, and more than 30% reported only surrogate outcomes. When a surrogate outcome is the only outcome of a drug trial presented, it is left to the individual clinician or authors of clinical guidelines to judge the clinical benefit of the medication. Our findings are consistent with other studies showing that surrogate outcomes alone often form the basis for drug approval. This was the case for nearly half of all new therapeutic agents approved by the U.S. Food and Drug Administration (FDA) during 2005–2013 [23]. In FDA drug approvals 2003–2013 for drugs used in COPD and diabetes, 78% and 100%, respectively, were based on surrogate outcomes alone, and only 25% of the approvals included a discussion of the scientific rationale for using surrogate outcomes [43]. This is concerning because a surrogate variable might not be a true predictor of the clinical outcome of interest, and might not provide a quantitative measure of clinical benefit that can be directly weighed against adverse events [44]. Sometimes an indirect measurement of an effect with a surrogate outcome is the only feasible possibility in a trial, but this should be justified in each case. We found that most trials presented modified intention-to-treat analyses. This type of analysis represents a post-randomisation exclusion of participants, potentially limiting the external validity [45]. Intention-to-treat analyses are generally recommended as a strategy for RCTs, although a strict intention-to-treat analysis may be difficult to achieve because of missing data or violations of the trial protocol [27]. However, whether the use of modified intention-to-treat analyses actually affects the intervention effect differs between studies [46, 47]. A recent meta-epidemiological study across therapeutic areas found that trials using a modified intention-to-treat strategy generally showed larger intervention effects than trials analysed using intention-to-treat analyses [46].

### Adverse events of treatment

As expected, we found that most trials reported adverse events; however, 7% did not report any clinical details of adverse events. Previous studies have shown that adverse events are often inadequately reported in journal articles compared with complete study reports [48]. We could not determine whether this was the case in our present study because we did not have access to the complete study reports. RCTs rarely assess harm as their primary outcome, and systematic reviews frequently report the harmful effects of an intervention inadequately [49]. A balanced reporting of benefits and harms related to an intervention is crucial. Many adverse events are first reported after longer-term use of the intervention than the trial period, which is often relatively short.

### Strengths and weaknesses of the present study

In the present study, we included all trials with published results from a complete national cohort of trials planned to be conducted partly in Norwegian general practice; however, there are possible limitations to the identification of the trials in the manual archive search and in the search for publications, as previously described [21, 22].

Several checklists for considering the reporting of aspects relevant for external validity have been proposed. We chose issues judged most relevant and feasible to consider. However, our selection may be discussed. We did not extract data regarding all possible aspects affecting external validity of the trials or all issues listed in Rothwell’s framework [1]. This would not be feasible for an entire cohort of trials from many different therapeutic areas. The choice of an active versus non-active comparator is particularly important for the assessment of external validity of a drug trial, and is covered by the domain “Differences between the trial protocol and routine practice” in Rothwell’s framework (Table 1) [1]. However, we found this to be beyond the scope of the present article.

Only one author extracted all data. Ideally, two authors should have coded all articles independently. It is therefore likely that some errors may have occurred during data extraction. However, we double-coded a random half to check quality and reliability, and the inter-rater agreement for this proportion was good as assessed by kappa, even though a critical appraisal of reporting involves several assessments. First, screened individuals may not be defined the same way in all articles. Consequently, there has been some room for judgement regarding these data. Further, for adverse events, we used a crude categorisation of reported/not reported. However, reporting of adverse events is not always a yes/no issue [48]. Finally, for surrogate outcomes, there are distinctions between outcomes recommended or not by various drug authorities [50]. However, we have not made distinctions between different surrogate outcomes in this article. Not all trials we assessed were RCTs; therefore, the heterogeneity of our study sample might be another limitation.

## Conclusions

We found that important external validity aspects were not always adequately reported in general practice drug trials. Some of these aspects are included in the CONSORT checklist, but despite this, frequently omitted, especially the reporting of clinical settings, but also the selection of patients before inclusion in a trial, which is emphasised in the CONSORT extension for pragmatic trials [7]. By contrast, other issues we consider important for external validity from a primary care viewpoint are lacking in the CONSORT checklist; for example, reporting of co-morbidity and co-medication of participants. These issues were often not reported, but encouragingly, we found that this reporting improved during our study period. Including these items among the reported clinical characteristics of trial participants would improve the assessment of the external validity of a clinical drug trial; therefore, we suggest specifying these issues in future revisions of the CONSORT checklist.

## Abbreviations

CENTRAL: Cochrane Central Register of Controlled Trials
CONSORT: Consolidated Standards of Reporting Trials
FDA: US Food and Drug Administration
GP: General practitioner
NoMA: Norwegian Medicines Agency
RCT: Randomised controlled trial
